# 

**Published:** 1892-08

**Authors:** 


### Southern Medical Record

A Monthly Journal of Medicine and Surgery.

VOL. XXII. ATLANTA, GA., AUGUST, 1892. NO. 8.

EDITORS:

A. W. GRIGGS, M. D. WILLIS F. WEST[illegible]

J. McFADDEN GASTON, M. D. DAN. H. HOWE[illegible]

TERMS: $2.00 Per Annum; Single Copy, 20 Cents.

Entered at the Postoffice at Atlanta, Ga., as Second-Class Matter.

Postell & Sexton, Publishers. 23 S. Broad, Atlanta, Ga.

